# Valedictory Address

**Published:** 1863-02

**Authors:** F. Mahla

**Affiliations:** Prof. of Chem. Med. Dep. Lind University


### CHICAGO MEDICAL EXAMINER.

N. S. DAVIS, M.D., Editor.

VOL. IV. FEBRUARY, 1863. NO. 2.

### Original Contributions.

### ARTICLE III.

### VALEDICTORY ADDRESS,

DELIVERED AT THE END OF THE WINTER-SESSION, 1862.

By F. MAHLA, A.M., Ph. D., Prof. of Chem. Med. Dep. Lind University.

Gentlemen of the Graduating Class:

You have devoted yourselves to an arduous profession, and have received the highest honor which could be conferred by our Alma Mater, under whose auspices you have begun and completed your studies.

It has been customary to select this occasion for commending to the attention of graduates, certain thoughts, which cannot very well be discussed in the ordinary course of instruction. The agreeable duty of following up this usage, at the end of the present term, has been assigned to me. In discharging this trust, I venture to say, that you are better prepared to commence your professional career, than the usual range of medical students. I do not say this with the intention to flatter your vanity, or to make an ill-timed compliment to ourselves, your late instructors. I believe, however, that you can rightfully claim a better qualification, on the ground, that the course of teaching in this Institution is so much more extended than that of other schools of medicine, that you have had ample occasion to devote your full attention to every branch of medicine. The clinical advantages, also, you have enjoyed, gave you rich opportunity to improve, and perfect your knowledge in every respect.

A new era in your life, Gents, commences to-morrow. You are going to show that the grain which you were sowing has developed itself to a plant, which bears fruit itself. When in the act of commencing such a new epoch in life, the human mind is more willing than ever to consider a friendly advice, as well as an earnest request. On this supposition, I wish to make you aware of the fact, that you assume this evening important responsibilities and duties. I need not enter into specifications, as far as your relations to the public are regarded, for it suffices to recommend you: try to be men in the noblest sense of the word, and you will readily discover the right ways. Be honest, upright, never faltering in the discharge of your professional services, and you will feel that you are doing justice to yourselves, as well as to the public.

I cannot omit, however, to bespeak at a greater length, a duty which you owe, not only to yourselves, but also to the noble profession to which you now belong. This important duty consists in continuing your studies with energy and perseverance. I pray you, therefore, Gents, not to throw your books away in leaving these halls; and let me hope that you will not forget this request. Alas, that so many who go forth from the various schools of medicine, suppose it to be sufficient if their fellow-citizens think them to be learned men, because they are permitted to append the M.D. to their autographs. The physician, who feels contented to possess an approximative perception of what concerns the human system, who feels exceedingly gratified if he can deceive others as to his real knowledge, that physician may attract patients, he may be able to amass wealth, but he is not worthy to practice the hypocratic art. He succeeds in a *certain* sense, but lacks the *true* success.

I can readily imagine, that some of you, in entering upon the study of medicine, have done it, not for the beauty of medical science, but for the seeming lucrativeness of the "business." Let me hope that none of you leave these halls with these unfortunate ideas, for I would feel that the instructions which you received in this Institution had been in vain. Indeed, very few physicians succeed in making fortunes by their practice; and, if you made wealth your aim, you may, perhaps, be sadly disappointed. I am certain, however, that *you* have higher motives, and that you love medicine not as a *business* but as a *science*.

You may have come to this school with the impression that medical knowledge consists in the art of getting up prescriptions. In leaving it now, you are aware that this part is of but minor importance, as far at least as the science of medicine is regarded. You know now, that this science is the means which enables you to solve even the most difficult questions that concern the vital system, yea, even life itself.

It is obvious that a science with so wide a field, requires deep and earnest studies. A man who ardently wishes to master his profession, must not wantonly pass over an imperfection in his knowledge. He must not fancy that the acquirement and cultivation of certain branch sciences of medicine is a burden, and will hinder his progress. Remember well, that a vessel which seeks deep water is precious and reliable; the shallow craft, however, though possessing speed, may capsize during the first gale, and woe then to the poor unfortunate who trusted to it too eagerly. Let me recommend you, therefore, to continue your studies; and, as far as I can see, you are not disposed to consider the granting of the title of Doctor of Medicine as the keystone of your professional education. Your education has, indeed, but just commenced, and it will take a long life of earnest study, to become adepts in your art.

Gents, you have chosen a noble avocation. BACON already said: "Physics is the mother of sciences, and contributes as such to the civilization of the people." History has approved of this sentence, and nothing is easier than to show the truth of these words. Let me merely remind you of past epochs, let me point out the times of the old powerful Roman Empire, when it was supposed to be necessary to create particular deities for every disease. Gods and goddesses were busy from the birthday of an individual up to his death-bed. The convulsive twitchings of a child, just as well as the accomplished mania of an adult, were ascribed to demoniac influences; and no cure was undertaken, unless the favor of the gods was bribed by immolations. Again, let me mention the times of the middle ages, when men were punished with death, under the pretence, they had by witchcraft produced epidemic diseases; and let me now direct your looks upon the enlightenment of the present age,—then you will ask with me: must we not feel gratified over the change that took place in so short a period? Civilization, like the rising sun, diffused its golden rays, until people bowed their heads in admiration of its power; and we may proudly say, that medicine has been the stimulus for its development.

In the course of time, when the healing art was no more inherited by tradition from one generation to the other, but made the object of regular studies, it was found that it had to derive its chief support from the socalled natural sciences.

Simple and natural, like the morals of the ancients, were, undoubtedly, also their medicaments. We know that the first medicines were derived from the vegetable kingdom. At a later period, when the necessity for iron tools was felt, the mineral kingdom was explored, and many mineral substances were soon also valued as remedial agents. The wants of the healing art were, however, daily increasing; and as soon as chemistry, in its first rudimentary form, made its appearance, its products were drawn into the circle of medicinal usefulness, and employed for the purposes of curing diseases. Prolongation of life, and the preservation of youth, were then the objects of research. Cosmetics, paints, and costly waters, made their appearance on the toilet-table, and were, probably, not less appreciated by the fair sex, than at present. The wheel was thus set into motion, and the most invaluable data were brought to light in rapid succession. Soon each of the natural sciences was made a special study. Chemistry especially, and physical philosophy, made such astonishingly rapid progress, that they had to be regarded not only as the fundamental branches of medicine, but soon became also, indispensable to industry and science. We see, thus, that the natural sciences, which were cultivated at first merely for the purposes of discovering means by which life might be prolonged, soon became the most effectual supports of refinement and welfare. Is this difficult to prove? If you think so, consider the single discovery of oxygen, made not a full century ago. The morals and habits of the whole civilized world were changed since that time. The knowledge of the composition of the atmosphere, that of the solid crust of the earth, of the water, and their influence upon animal and vegetable life, are connected with it. The lucrative running of innumerable factories and trades, the important metallurgic processes, by which the precious metals can be extracted from the earth, depend on it. Thus, it may be asserted, without hesitation, that the material welfare of entire nations has been doubled and quadrupled since that discovery; and I do not believe we say too much, in proclaiming, that the wealth of every individual has been increased by it.

Every discovery brought to light by studying natural sciences, has similar beneficent consequences, and affords advantages to the individual as well as to the community, by increasing their power and well-being. In order to convince you of this fact, look at more recent discoveries. Not many years ago, the world was startled by the invention of the iron horse; soon after, flashes of lightning were used to send a friendly word from one hemisphere to the other; still later, the solar light was employed, not only by the artist, who painted with its fiery pencils, but also by the chemist, who detected with its aid, traces of substances, in proportions, which a mathematician will find difficult to calculate. All this has been accomplished by those natural sciences, which received the first impulse for their development through the study of medicine. But what has become of this latter science itself? Has it made equal progress with its sister sciences? It is true, medicine also has been developed astonishingly. Talented men devoted their energies to it; but if we disregard the wonderful discoveries in surgery, must we not acknowledge that a great deal remains to be done in the province of medicine, before empyricism shall make room for a rational and positive science?

I could mention numerous facts, which would convince you of the truth of my assertion. A few, however, will suffice, and I refer, therefore, to the struggle between solidism and humorism. If you carefully consider the principles of these two theories, you will discover that the idea of the personification of disease is put forth by them. Disease was regarded, during centuries, as something which had existence in itself, and was, as such, contrasted with health. The more this idea gained ground, the more appeared the necessity to locate, at least, disease in certain parts of the animal organism. Solidism suggested that it resided especially in the solid parts of the body, while humoral pathology argued its existence in the fluids. Involuntarily, a conflict was thus originated, in which the partizans of either side had the same watchword: here disease, there health and life. Life and disease carried on war in the body, against each other. The result of this supposed struggle was, however, always unfavorable to disease, for its victory was a suicide, in as far as it perished with life itself. This contradiction should have been sufficient to show the incorrectness of the idea. Indeed, it does not seem difficult at all to see that disease is nothing else than life itself, whose functions, owing to the change of outside influences, are manifested in an exceptional form. This view of the nature of diseases is even not shaken by the sudden appearance of epidemics; for, if disease has to be regarded as the irregular manifestation of an individual life, we have to look upon epidemic diseases as upon the symptoms of a disturbance in the life manifestations of entire masses. They will make their appearance when and wherever numbers of people are subjected to the same unfavorable circumstances.

The vehement and dreadful suddenness, with which epidemic diseases often made their appearance, had, during centuries, the effect of almost paralyzing the human intellect. Instead of searching for *natural* explanations, the fundamental indolence of mankind found it easier to attribute to them a divine origin; and their existence in certain localities, was only too often called a divine punishment for human sinfulness.

If, however, disease is nothing else but life under changed and unfavorable conditions, then it is obvious that curing disease is nothing else but the restoration or re-establishment of the normal life-manifestations.

Life is not a condition which remains the same at all times, but one which continuously *re*forms. We may compare it with a river, which uninterruptedly rolls away in the individual channel; and the highest duty of the medical man, consists in regulating its current, removing obstructions from its course, so that it cannot cause destruction by overflowing, nor loose itself among sands.

Modern medical science refutes the phantom of a personified disease. Let us do the same, and we will find that we have to drop also the idea of the existence of specific remedies. We strip the latter of their magic charm, by destroying the entity of diseases.

It is not very long ago, that doubts as to the existence of specifics, were timidly raised. Mercury in syphilis, quinine in fevers, were quoted as such. We know now, that not diseases as such are the objects of therapeutics, we regard, as such, merely the changed and irregular manifestations of life; and we must admit, that these can but very seldom be identical. This single quotation already might convince you, that the science of medicine is daily undergoing transformation. I might mention hundreds of similar instances; and I entertain no doubt they would have the effect of rousing not only your interest, but also your ambition, to participate in solving these seeming mysteries.

Some of you might, however, be inclined to say: true, had we been living at that time, we also would have contributed our share, but these questions and uncertainties are now fully settled. To those I would only suggest, that the field of observation is daily getting broader and wider. Not long ago, our science only recorded the positive fact of a source of continuous heat in the animal organism, without attempting a further explanation; when the discovery of oxygen and LAVOISIER's theory, made it possible to view the respiratory process as a combustion. The suggestion that animal heat was the natural consequence of this combustion process, was near enough, and also this problem seemed to be fully settled. The assumption was made, that the stability of animal heat was maintained by the combustion of carbon in the body; and even our most celebrated men asserted, singularly enough, that by this combustion, just as much heat was developed as by the combustion of carbon in air or in oxygen: and the attempt was even made to show by calculation the necessity of this stability of heat. Soon, however, the suggestion was raised, that the comparison between the two combustion processes had to be essentially modified; for we have not carbon in the state of an element in the system, but as a constituent of those numerous organic combinations which form component parts of the animal body. Many of these contain, besides carbon, hydrogen and oxygen, also nitrogen, and still others in addition, sulphur and phosphorus; and it is obvious that all these substances are not only liable, but must undergo, simultaneously, combustion. This combustion does, however, not take place in such a manner that the final products which we exhale or secrete are generated directly by the entire dissolution of the muscular substance, or similar constituents of the organism. It is now a well-established fact, that many intermediate compounds are generated, before the final products—carbonic acid, urea and water—are ejected as effete matters from the system.

Alcohol, *f. i.*, is first converted into acetic acid, this latter into oxalic acid, before carbonic acid and water are produced from it; and how numerous are the intermediate products of combustion, generated from such complicated bodies as albumen, fibrin, etc., before they are resolved into urea, carbonic acid, and water?

Finally, it seems very likely that this complicated combustion is not the only source of heat. How much of it may be attributed to other chemical processes, as *f. i.*, to the chemical combination of acids and bases, or the absorption of gaseous substances in liquids, is difficult to say.

One thing is, therefore, certain, that the original view as to the source of heat, was not correct; and that the natural philosophers in attempting to explain by calculation the stability of heat, and taking carbon only in consideration, committed a gross mistake. Animal heat is but one of those numerous vital phenomena, for whose elucidation the physiological chemist studies all those minutiæ, and retains them in his memory. For whom, indeed, could the study of chemistry and chemical physiology be of a greater interest than the medical man? for whom could it be more profitable and necessary to gain an insight into the processes of digestion, respiration, and nutrition? and, still it is a fact, that this beautiful science which constitutes, in reality, the basis of medicine, is usually neglected by the physician. It is true, the attention of medical men in Europe has, during the last decennia, been more and more directed towards this study, for the development of physiology made it an absolute necessity. It seems to be different, however, on this side of the ocean.

The people of these States are said to be money-loving; and calumniators dared even to assert they placed the money-making principles above their God. If ever this should have been so, let me hope that this period has passed by, especially in medicine. There may have been, or there are, perhaps, physicians, who know medicine, not as a science, but as an *experimenting* art, who recognize no principles, but rules taught by experience, who suppose it to be sufficient if they remember how this or that operates in this or that case. Such an experimenting art does not inquire into reasons, it ignores the little word "why." Without a correct conception of natural phenomena, without a thorough physiological and chemical education, is it to be wondered that such physicians change so easily their professional creeds?

The doctrines of HAHNEMAN found, at a certain period, followers in Germany, in that land in which they were originated; but science did not long tolerate the ridiculous and pigmean excrescence. Homœopathy had to die, before the swaddling-clothes were fairly removed from the ill-born creature.

The seeds of useless weeds, however, are easier propagated than those of cereals. We are all aware of this fact, and it may serve us to explain the strange phenomenon, that doctrines which were repudiated in the country that originated them, found a fertile soil on this side of the Atlantic. Our great chemist, LIEBIG, in alluding *to the same thing*, says: "Intelligence alone, does not protect even entire nations from aberrations of mind; the child, however, ceases to fear phantoms, the more its mental faculties and knowledge are developed." Gents, I think we may console ourselves with him.

In entering now, Gents, upon your professional career, you will meet strange brethren. You will find not only partizans of all sorts of isms, you will also encounter men who pretend having made regular studies, but deport themselves as if they had discovered the philosopher's stone, and were infallible in their skill. To hear this sort of men, they never fail to cure a disease, and always send their patrons away rejoicing. Such men are usually good business managers, they are shrewd and sharp, and never hesitate to take advantage of even the vilest and most despisable means. Every wheel is set into motion by them, in order to gain notoriety. They, *f. i.*, not only make advertisements, by covering all buildings with immense posters; they also manage, in some way or the other, to have an editorial friend, who, from time to time puffs, in an extra item, the wonderful cures of the doctor. Usually, also, they make a special disease their favorite; and all their patients must be afflicted with that speciality. To hear them, they have, *f. i.*, any numbers of patients with typhoid, or with heart disease, and these they cure in less than no time.

I need not warn you to avoid this sort of proceeding. You are too well-versed in the science of medicine, to sink to the rank of charlatans.

I cannot omit, however, to refer to others, whose moral principles are not superior to those of the preceding. There are physicians, who adopt the policy to see in every little indisposition a grave disease, in order to cover themselves with glory, by showing how rapidly they can cure it. Shall I urge you, Gents, to avoid also this reef? If you consider that your opinion as to the probable result of an indisposition is often regarded as authority, if you perceive what amount of grief and sorrow you are able to produce in already afflicted hearts, *you* certainly do not wish to earn your fame in this way. Still worse in his acts is, however, that medical man, who, in preferring the surgical arena, never hesitates to perform an operation, though he is well aware, that by undertaking it he sacrifices the life of his patient. He is doing it, however, with the hope to acquire thus the renown of a skilful operator.

In cautioning you not to imitate these men, I do not intend to advise you to hide yourselves before your fellow-citizens. Times will come, when your firm hand will prove you a good operator, when your diagnosis will herald you a physician, and will bring you fame and success.

Success! What a charm for a beginning practitioner, is in the few words: professional success! Honor and wealth are coupled in it. Many who find the first, cannot get the latter; few can boast of having acquired both; but still fewer can pride themselves of true success. By true success, I do not mean the accumulation of riches, for they may be also, and in most cases are, quicker obtained by the mere charlatan. I mean by it, that success which every man finds in the faithful discharge of his duties, which his own conscience is granting him; and allows him, when more advanced in years, to review, with pleasant feelings, his former life.

Gents, do you wish a success like this? If so, you must throw aside all selfish thoughts, must never complain of tiresome professional duties, and must possess a great deal of self-sacrificing energy. The life of such a physician is, however, far from being always pleasant; for you may be summoned at nights, or at daytime, you must never hesitate to follow. May it be the costly mansion of the rich, or the humble homestead of the poor, go and bring them relief if you can. If then, in after years you can say, that you were able to dry the flowing tears, and fill with joy again the sorrowed hearts, by your professional skill, then you will grant the truth of my assertion: this only was a true success. You will, then, esteem yet higher, the noble profession which you have chosen for your career.

Gentlemen, the course of instruction, which this evening ceases, has given us occasion to form an acquaintance with each other, which, as I hope, will not lack of future friendship.

In commencing your professional life, I am confident you will give credit to the Faculty of the Medical Department of Lind University, for its efforts to make your attendance in this Institution both agreeable and profitable. You received the Diploma of this School of Medicine, as a just tribute of your medical attainments. As I know you, I feel confident you will continue your studies, you will love and honor your profession, you will rank high in all moral perfections, in fact, you will acquit yourselves like men.

I cordially tender you, therefore, in the name of our Faculty, my hand, and bid you a friendly and affectionate farewell.

